# A phase II, sham-controlled, double-blinded study testing the safety and efficacy of the coronary sinus reducer in patients with refractory angina: study protocol for a randomized controlled trial

**DOI:** 10.1186/1745-6215-14-46

**Published:** 2013-02-15

**Authors:** E Marc Jolicœur, Shmuel Banai, Timothy D Henry, Marc Schwartz, Serge Doucet, Christopher J White, Elazer Edelman, Stefan Verheye

**Affiliations:** 1 Montreal Heart Institute, Université de Montréal, 5000 Bélanger Street East, Montréal, Québec Q H1T 1C8, Canada; 2 The Tel Aviv Medical Center, The Tel Aviv University Medical School, 6 Weizman Street, Tel Aviv, 64239, Israel; 3 Minneapolis Heart Institute Foundation at Abbott Northwestern Hospital, 920 East 28th Street, Minneapolis, MN, 55407, USA; 4 Neovasc, Inc., 137000 Mayfield Place, Richmond, BC V6V 2E4, Canada; 5 The John Ochsner Heart & Vascular Institute, Ochsner Clinical School, The University of Queensland School of Medicine, 1514 Jefferson Highway, New Orleans, LA, 70121, USA; 6 Harvard-MIT Division of Health Sciences and Technology, 77 Massachusetts Avenue, Cambridge, MA, 02139, USA; 7 Cardiovascular Division Brigham and Women’s Hospital, Harvard Medical School, 75 Francis Street, Boston, MA, 02115, USA; 8 Antwerp Cardiovascular Institute, ZNA Middelheim Hospital, Lindendreef 1, 2020, Antwerpen 22, Belgium

**Keywords:** Angina, Refractory angina, Advanced coronary artery disease, Myocardial ischemia, Coronary sinus, Reducer

## Abstract

**Background:**

A growing population of patients lives with severe coronary artery disease not amenable to coronary revascularization and with refractory angina despite optimal medical therapy. Percutaneous reduction of the coronary sinus is an emerging treatment for myocardial ischemia that increases coronary sinus pressure to promote a transcollateral redistribution of coronary artery in-flow from nonischemic to ischemic subendocardial territories. A first-in-man study has demonstrated that the percutaneous reduction of the coronary sinus can be performed safely in such patients. The COSIRA trial seeks to assess whether a percutaneous reduction of the coronary sinus can improve the symptoms of refractory angina in patients with limited revascularization options.

**Methods/Design:**

The COSIRA trial is a phase II double-blind, sham-controlled, randomized parallel trial comparing the percutaneously implanted coronary sinus Reducer (Neovasc Inc, Richmond, BC, Canada) to a sham implantation in 124 patients enrolled in Canada, Belgium, England, Scotland, Sweden and Denmark. All patients need to have stable Canadian Cardiovascular Society (CCS) class III or IV angina despite optimal medical therapy, with evidence of reversible ischemia related to disease in the left coronary artery, and a left ventricular ejection fraction >25%. Participants experiencing an improvement in their angina ≥2 CCS classes six months after the randomization will meet the primary efficacy endpoint. The secondary objective of this trial is to test whether coronary sinus Reducer implantation will improve left ventricular ischemia, as measured by the improvement in dobutamine echocardiogram wall motion score index and in time to 1 mm ST-segment depression from baseline to six-month post-implantation.

**Discussion:**

Based on previous observations, the COSIRA is expected to provide a significant positive result or an informative null result upon which rational development decisions can be based. Patient safety is a central concern and extensive monitoring should allow an appropriate investigation of the safety related to the coronary sinus Reducer.

**Trial registration:**

ClinicalTrials.gov identifier - NCT01205893.

## Background

In patients with stable coronary artery disease (CAD), a successful treatment is defined as a complete, or nearly complete, elimination of anginal chest pain with improved functional class and a return to normal activities [1]. This goal cannot be achieved in a growing population of patients [2,3] with advanced CAD having no option for further revascularization either by percutaneous coronary intervention (PCI) or by coronary artery bypass graft (CABG) surgery. These ‘no option’ patients remain severely disabled by chronic refractory angina pectoris, despite optimal medical therapy. The worldwide prevalence of ‘no option’ patients with refractory angina is growing and new therapeutic options are required [4].

Experimental evidence revealed that in the setting of a significant epicardial coronary artery stenosis, increased coronary sinus (CS) pressure can lead to redistribution of blood flow from nonischemic to ischemic myocardial territories [5]. In the 1950s, partial ligation of the CS was hypothesized to force oxygenized arterial blood into underperfused myocardial segments^2^. Historically, the partial ligation of the CS by surgery has been associated with a reduction in mortality (13% vs. 30% for nonoperated subjects) [6,7], and an improvement of angina [6-10]. The success of CABG decreased interest in surgical ligation of CS but the current rise in patients with refractory angina creates an opportunity to reconsider this approach.

In the healthy heart, selective sympathetically mediated constriction of subepicardial vessels during exercise maintains adequate subendocardial perfusion and a proper myocardial function. In the presence of an epicardial coronary artery stenosis, the constriction of subepicardial vessels becomes dysfunctional and is unable to compensate for the lack of oxygenated blood [11]. In patients with ischemic cardiomyopathy, the perfusion of the subendocardial layers may be further compromised whenever the left ventricle end-diastolic pressure is elevated [12]. In the setting of advanced CAD, elevated CS pressure could ameliorate subendocardial ischemia by normalizing the subendocardial to subepicardial blood flow ratio and lead to redistribution of collateral blood flow from nonischemic to ischemic territories of the myocardium.

### Percutaneous CS reduction

**Figure 1:**
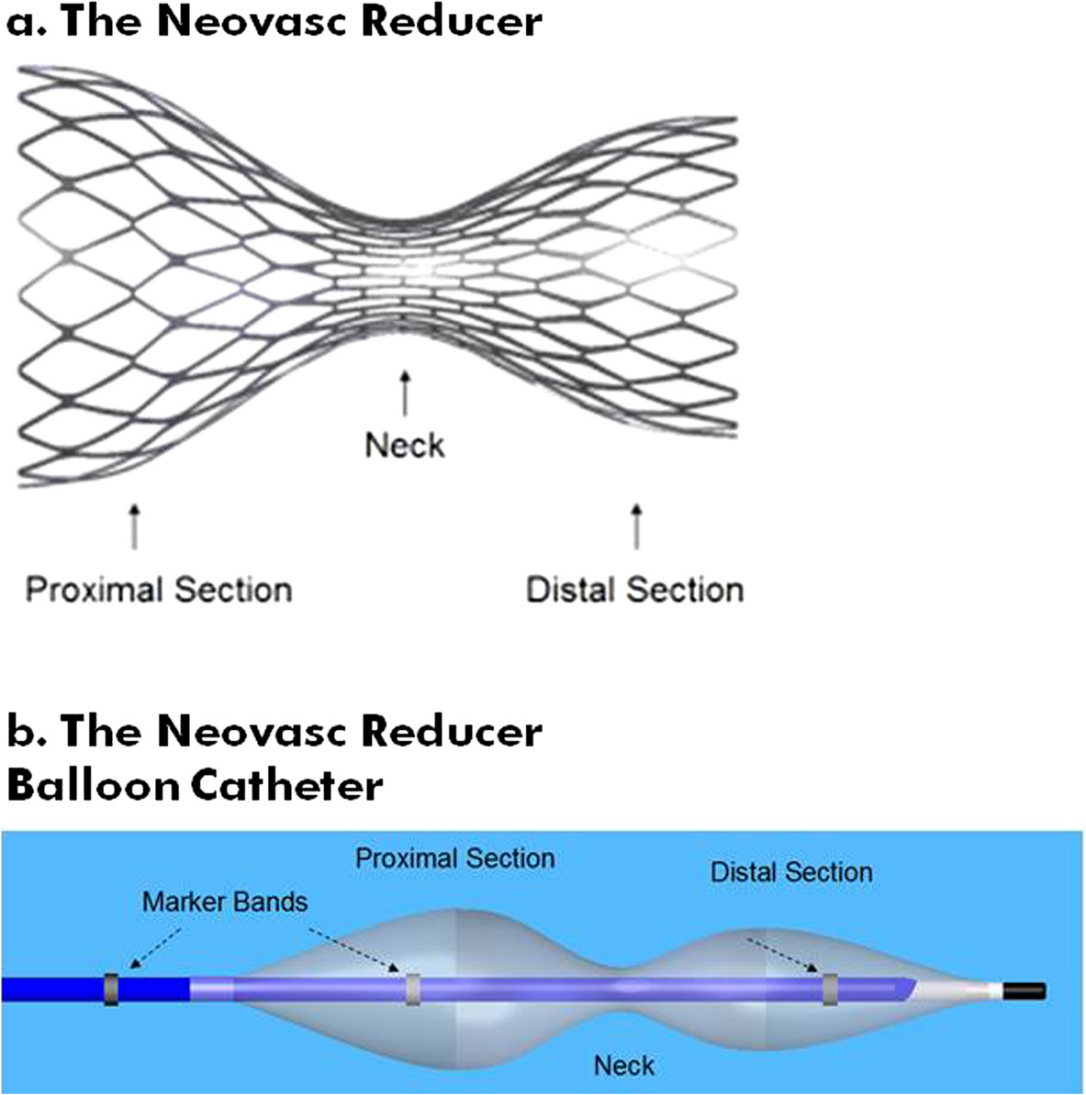
**The Neovasc Reducer**™ **System. **The Neovasc Reducer™ System is comprised of the Neovasc Reducer™ premounted on the Neovasc Reducer™ Balloon Catheter. **(a) **The Neovasc Reducer. **(b) **The Neovasc Reduce Balloon Catheter.

Today, the concept of percutaneous CS narrowing is emerging as a possible treatment option. The coronary sinus Reducer (Neovasc Inc, Richmond, Canada) is a percutaneous, endoluminal, hourglass-shaped, balloon-expandable stainless steel device that is designed to be implanted in the CS to create a controlled local narrowing, which modifies myocardial blood flow and coronary sinus pressure (Figure 1a). By increasing the CS pressure, the Neovasc Reducer™ System is thought to favor redistribution of oxygenated coronary artery blood flow toward underperfused ischemic subendocardium. The Reducer is made of surgical grade 316LVM stainless steel, laser cut into a prespecified geometric pattern with flexible longitudinal struts and no welding points. The Reducer is available in one single model designed to fit a range of anatomies. Its final expanded diameter is dependent on the inflation pressure of the semi-compliant balloon. The Neovasc Reducer™ Balloon Catheter (Figure 1b) is an over-the-wire catheter with a unique hourglass-shaped balloon to conform to the tapering typically encountered in the CS.

In 2007, Banai *et al*. reported first-in-man experience with the Reducer in 15 unrevascularizable patients [13]. In 12 patients, the Reducer was associated with a significant improvement in angina class (Canadian Cardiovascular Society (CCS) scores 3.07 at baseline vs. 1.64 at follow-up, *P* <0.0001). The symptom relief correlated with objective myocardial ischemia reduction measured with thallium single-photon emission computed tomography (SPECT) and stress echocardiography. At three-year follow-up, no patient experienced death or myocardial infarction attributable to the device. The computed tomography (CT) angiography revealed patency of all Reducers with no evidence of device migration. Overall, the improvement in angina score was maintained [14].

The supportive experimental evidence combined with first-in-man results led to the Coronary Sinus Reducer for Treatment of Refractory Angina (COSIRA) trial, which is designed to provide an alternative treatment strategy to improve symptoms of refractory angina in no option patients with reversible myocardial ischemia, secondary to CAD.

## Methods/Design

### Study objectives

COSIRA is a prospective, multicenter, randomized, double-blind, sham-controlled clinical trial of the safety and effectiveness of the CS Reducer. The primary endpoint of this trial is improvement in angina symptoms, measured with the CCS classification, in patients with limited revascularization option despite severe refractory angina. This study also aims to demonstrate that the Reducer implantation can be performed safely with a minimally invasive percutaneous approach. Other secondary objectives of this trial will test whether CS Reducer implantation will improve myocardial ischemia, as measured by a sestamibi (MIBI) SPECT stress test, the dobutamine echocardiogram wall motion score index, the Seattle Angina Questionnaire (SAQ) and the time to 1 mm ST-segment depression.

### Study population and patient selection

One hundred and twenty-four patients are currently being enrolled at up to 14 sites located in Canada, Belgium, England, Scotland, Sweden and Denmark. Inclusion and exclusion criteria are presented in Table 1. Study participants must experience CCS class III or IV angina pectoris despite attempted optimal medical therapy for 30 days prior to screening. Optimal medical therapy includes beta-blockers, calcium-channel blockers or long-action nitrates used at maximal tolerable doses [15]. Only patients with limited coronary revascularization options, either by PCI or CABG, are eligible to participate in the study. The details related to the unsuitability for revascularization are reviewed in details elsewhere [16].

**Table 1:** Inclusion and exclusion criteria for COSIRA

**A. Inclusion criteria**	**B. Exclusion criteria**
1. Patient >18 years of age	**Clinical**
2. Symptomatic CAD with chronic refractory angina pectoris classified as CCS class III or IV despite attempted optimal medical therapy for 30 days prior to screening	1. Recent (<3 months) acute coronary syndrome
3. Patient has limited treatment options for revascularization by CABG or PCI	2. Recent (<6 months) successful PCI or CABG
4. Evidence of reversible ischemia that is attributable to the left coronary arterial system by dobutamine echocardiography	3. Recent (<1 month) unstable angina (recent onset, crescendo, or rest angina with ECG changes)
5. Left ventricular ejection fraction >25%	4. De-compensated CHF or hospitalization due to CHF during the 3 months prior to screening
6. Male or nonpregnant female (NB: Females of child-bearing potential must have a negative pregnancy test)	5. Patient with pacemaker or defibrillator electrode in the right atrium, right ventricle, or coronary sinus
7. Patient understands the nature of the procedure and provides written informed consent prior to enrollment	6. Life-threatening rhythm disorders or any rhythm disorders requiring an internal defibrillator and or pacemaker
8. Patient is willing to comply with specified follow-up evaluation and can be contacted by telephone	7. Severe COPD as indicated by a forced expiratory volume in one second <55% of the predicted value
	8. Patient cannot undergo exercise tolerance test (bicycle) for reasons other than refractory angina
	9. Severe valvular heart disease
	10. Patient having undergone tricuspid valve replacement or repair
	11. Chronic renal failure (serum creatinine >2 mg/dL), including patients on chronic hemodyalisis
	12. Moribund patients, or patients with comorbidities limiting life expectancy <1 year
	13. Contraindication to required study medications that cannot be adequately controlled with premedication
	14. Known allergy to stainless steel or nickel
	15. Currently enrolled in another investigational device or drug trial that has not completed the primary endpoint or that clinically interferes with the current study endpoints
	**Anatomical**
	16. Mean right atrial pressure ≥_ 15 mmHg
	17. Patient with anomalous or abnormal CS as demonstrated by angiographic abnormalities defined either:
	a. Abnormal CS anatomy (for example, tortuosity, aberrant branch, persistent left SVC) and/or;
	b. CS diameter at the site of planned Reducer implantation <9.5 mm or >13 mm

*CABG *coronary artery bypass graft; *CAD *coronary artery disease; *CCS *Canadian Cardiovascular Society; *CHF *congestive heart failure; *COPD *chronic obstructive pulmonary disease; *CS *coronary sinus; *ECG* electrocardiogram; *PCI *percutaneous coronary intervention; *SVC* superior vena cava.

Before randomization, participants undergo a history to determine their CCS angina class, a physical examination, a baseline SPECT stress test, and a dobutamine stress echocardiogram. The day of the planned intervention, all participants undergo a right heart cardiac catheterization and a CS angiogram. Only candidates showing a mean right atrial pressure ≤15 mmHg with CS anatomy suitable for the Reducer implantation (nontortuous, nonaberrant, CS diameter >9.5 mm but <13 mm) are eligible for randomization.

The final protocol and amendments as well as the consent form are reviewed and approved by the institutional review board and independent ethics committee at each participating center. The study is being conducted in compliance with the provisions of the Declaration of Helsinki and relevant local country regulations. All patients must provide written informed consent.

### Randomization and treatment protocol

Participants are randomized in a 1:1 ratio using a centrally controlled computer-generated random allocation sequence. Treatment assignments are concealed in numbered sealed envelopes. The allocation sequence remains concealed until the study arm is assigned. Once randomization is assigned, the participant is officially enrolled in the study [17].

All participants remain blinded throughout the six-month study period. While the interventional cardiologist implanting the Reducer is not blinded, the study participants, the physician investigator responsible for assessing the CCS angina class at follow-up, all core laboratories, the biostatisticians performing the analysis, the members of the Clinical Event Committee (CEC), as well as the members of the Data Safety Monitoring Board (DSMB) are blinded to treatment assignment, which will remain until study completion and until after the database has been locked.

### Coronary sinus reduction

All participants are pretreated with ^3^ 80 mg aspirin daily for at least 72 hours prior to the device implantation with either clopidogrel (75 mg daily for at least seven days prior to the procedure or loading dose of 300 to 600 mg within 24 hours prior to the procedure) or prasugrel (loading dose of 60 mg within 24 hours prior to the procedure) continued for six months. Participants randomized to the Reducer undergo systemic anticoagulation with either an initial heparin bolus (70 U/Kg) to reach and maintain an activated clotting time above ≥200 seconds, or with bivalirudin (initial bolus 0.75 mg/Kg, drip 1.75 mg/Kg/hr).

**Figure 2:**
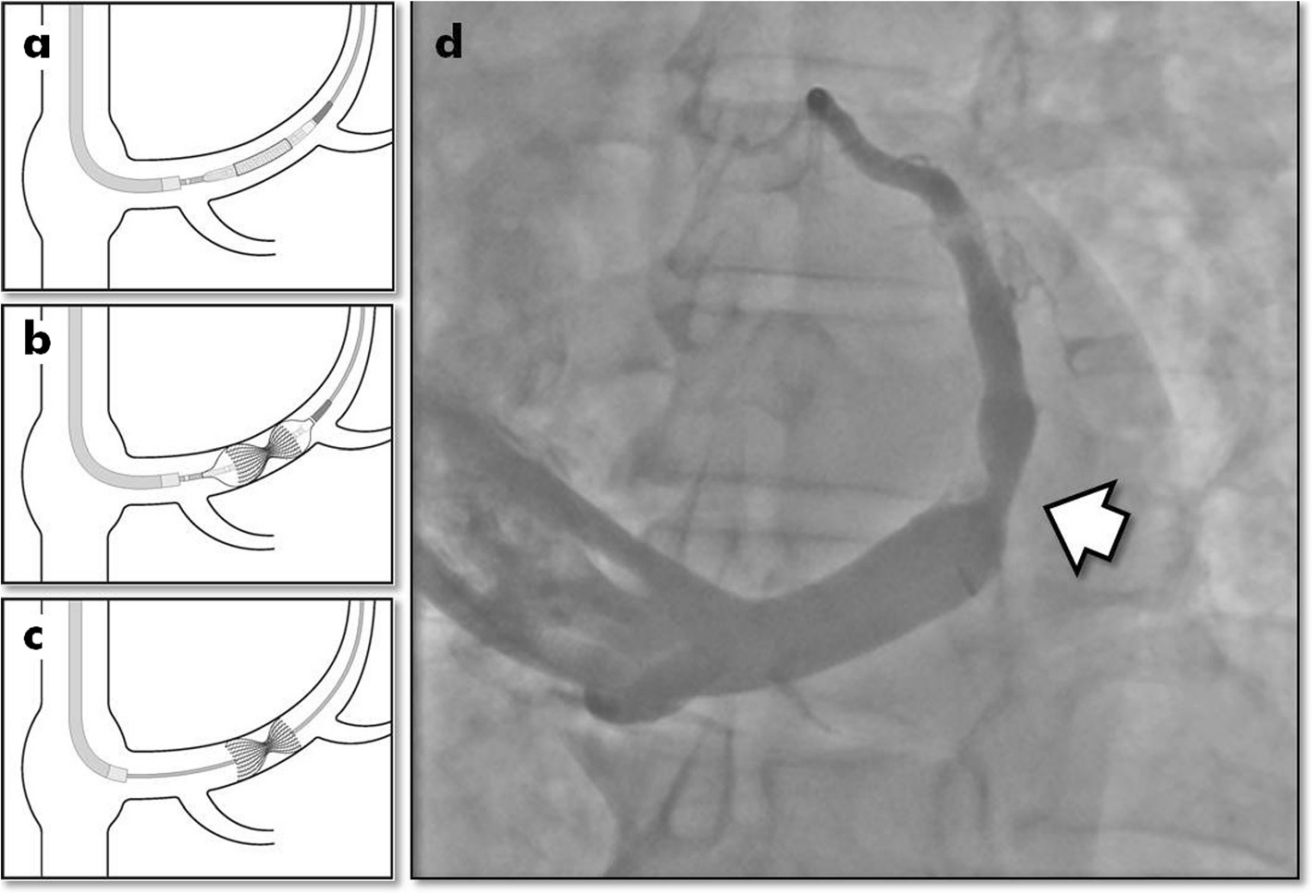
**Delivery and implantation of the coronary sinus Reducer. **Schematic representation of the delivery **(a)**, dilation **(b) **and implantation **(c) **of the Reducer in the coronary sinus. The Reducer has the appearance of an hourglass (arrowhead) once fully implanted **(d)**. Immediately after the implantation, the sinus remains patent and functional.

The methods of device implantation have been described previously [13] using a percutaneous jugular transvenous approach (Figure 2). Patients randomized to the Reducer group have a Reducer implanted immediately following the CS angiography. Once the CS is cannulated, a Reducer implantation takes approximately 15 minutes.

Participants assigned to the control group will undergo a sham procedure, but with no additional invasive manipulation than those required during the qualifying right heart catheterization. The implanting physicians are instructed to behave similarly during both Reducer and sham implantations. Participants are offered headsets playing music to mask the surrounding noise resulting from the intervention and conscious sedation per local practice.

### Outcomes

Primary and secondary endpoints of the COSIRA trial are listed in Table 2. The primary end points will compare the number of participants experiencing angina improvement ≥2 CCS grades six months after the intervention. To maintain blinding, baseline and six-month CCS grading are done by independent physicians unaware of the treatment allocation. Questions to the patients about their condition, with regards to CCS scoring, are scripted to ensure that every physician asks the same questions in an identical manner to each patient.

**Table 2:** Outcomes of the PRIMACY trial


**Primary endpoints**	1. Proportion of patients experiencing an improvement of 2 or more CCS angina classes.
**Secondary endpoints** The secondary endpoints will compare the variation of the change from baseline to 6-month follow-up:	1. Wall motion score index by dobutamine stress echocardiogram
	2. Exercise treadmill test
	a. The variation (change from baseline) in time to 1 mm ST-segment depression (min);
	b. Total exercise duration (min);
	c. Maximal ST-segment depression (mm)
	3. Proportion of patients experiencing an improvement of ≥ _ 1 CCS angina classes
	4. MACE - the composite of cardiac death, major stroke, and myocardial infarction
	5. The Seattle Angina Questionnaire scores, subdivided in categories
	a. physical limitation;
	b. anginal stability;
	c. anginal frequency;
	d. treatment satisfaction, and
	e. disease perception
	6. Reduction in reversible perfusion defect by SPECT
	7. Regional oxygenation improvement measured by BOLD CMR (in selected centers only)

*BOLD *blood oxygen level-dependent; *CCS *Canadian Cardiovascular Society; *CMR *cardiac magnetic resonance; *MACE *major adverse cardiac event; min, minutes; mm, millimeters; *SPECT *single-photon emission computed tomography.

**Figure 3:**
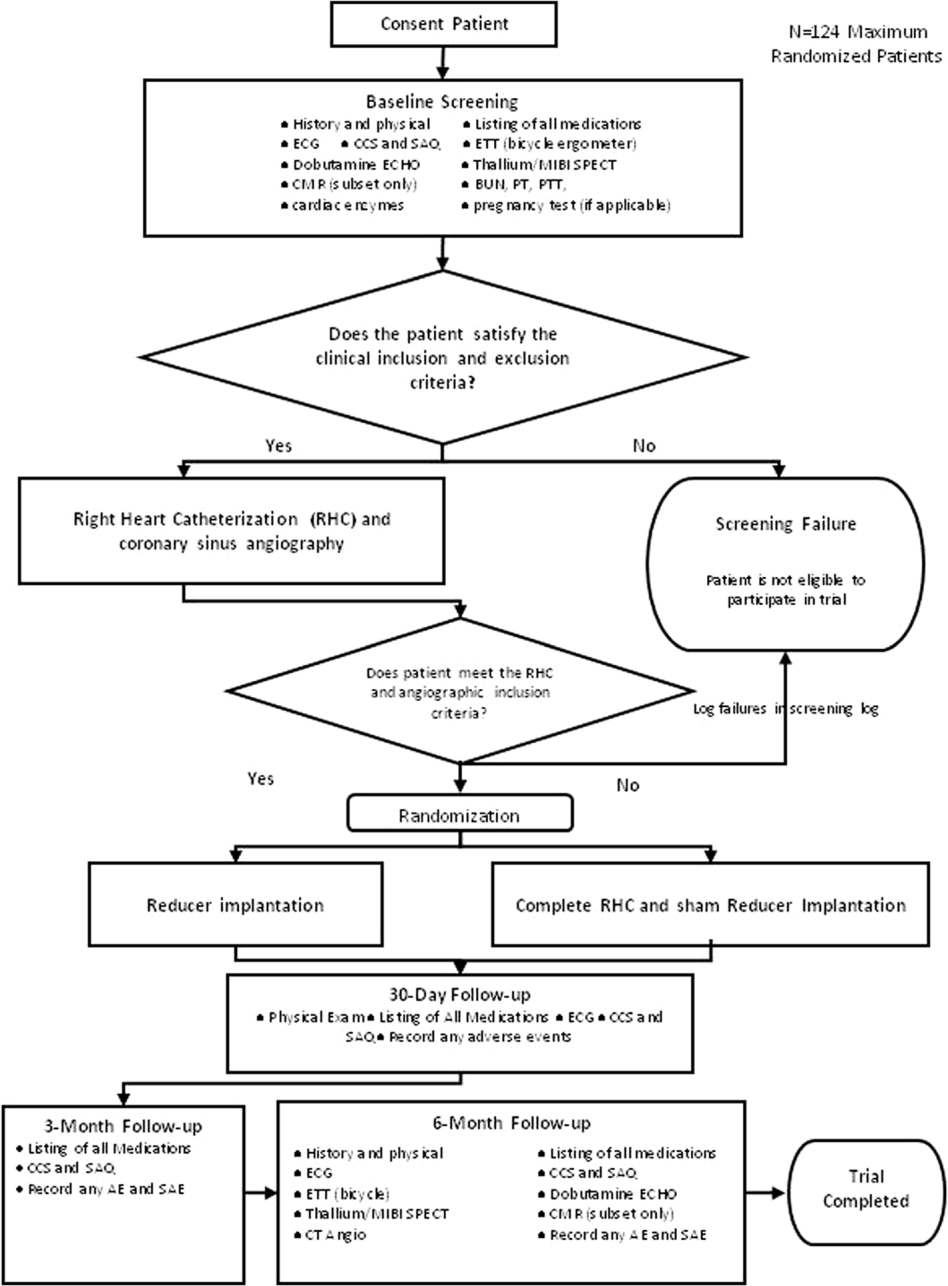
**COSIRA study flow. **AE, adverse event; CCS, Canadian Cardiovascular Society; CMR, cardiac magnetic resonance imaging; CT, computerized tomography; ECG, electrocardiogram; ECHO, echocardiogram; ETT, exercise tolerance test; MIBI, sestamibi; RHC, right heart catheterization; SAE, serious adverse event; SAQ, Seattle Angina Questionnaire; SPECT; single-photon emission computed tomography.

After the procedure, participants are assessed at hospital discharge, and post-procedurally at 30 days (office), three months (office or telephone), and six months (office) (Figure 3). At each of these follow-up visits, the occurrence of adverse events is evaluated. At the six-month visit, an exercise tolerance test, a SPECT stress test, and a dobutamine echocardiogram are performed. Following the final safety and efficacy assessments, all participants randomized to the Reducer undergo a CT angiogram to assess CS patency. The schedule of procedures for the trial is presented in Table 3.

**Table 3:** Study procedures

	**Screening**	**Procedure**	**Discharge**	**30 Days**	**3 Months**	**6 Months**
**Procedure**						
Informed consent	X					
Medical history	X					
Physical exam	X		X	X		X
Listing of medication	X		X	X	X	X
Pregnancy test	X^1^					
Blood work	X^1^					
Cardiac enzymes	X^2^		X^4^			
ECG	X^2^		X	X		X
CCS assessment	X			X	X	X
ETT	X					X
Dobutamine echo	X					X
Thallium/MIBI SPECT	X					X
SAQ	X			X	X	X
CMR	X					X
RH catheterization		X				
CS angiography		X				
Randomization		X				
Reducer implantation		X^3^				
CT angiogram						X^3^
Adverse events		X	X	X	X	X
Serious adverse events		X	X	X	X	X

^1^Within seven days prior to procedure; ^2^within 24 hours prior to procedure; ^3^in Reducer group only, to be performed only after final assessments CCS angina class and SAQ; ^4^cardiac enzymes (CK, CK-MB, and troponin) within 8 to 12 hours post-procedure. *CCS *Canadian Cardiovascular Society; *CMR *cardiac magnetic resonance imaging; *CS *coronary sinus; *CT *computerized tomography; *ECG* electrocardiogram; *ETT* exercise tolerance test; *MIBI *sestamibi; *RH *right heart; *SAQ *Seattle Angina Questionnaire; SPECT single-photon emission computed tomography.

A bicycle ergometry stress test adapted from the Asymptomatic Cardiac Ischemia Pilot (ACIP) protocol [18] was selected because the incremental increases in exercise workload are more gradual (≤1.5 METS/stage) compared to the larger work demands inherent in other exercise testing protocols (for example, the Bruce protocol). In patients with advanced coronary artery disease, this approach provides a greater discrimination in defining the time to onset of 1 mm ST-segment depression, angina, and heart rate increase [19]. Exercise stress test data as well as the dobutamine stress echocardiography will be extracted and interpreted by an independent core laboratory blinded to treatment assignment. Similarly, data for the dobutamine stress echocardiography wall motion score index (WMSI) will be extracted and interpreted by an independent core laboratory blinded to treatment assignment.

The Seattle Angina Questionnaire (SAQ) score [20], a brief 19-item self-administered questionnaire that captures five perspectives: physical limitation, anginal stability, anginal frequency, treatment satisfaction, and disease perception will be used to assess quality of life.

The safety of the Reducer implantation will be monitored by the procedural success, defined as successful delivery and deployment to the intended site in the absence of an adverse or serious adverse device-related event prior to hospital discharge. Periprocedural serious adverse events are defined as a composite of death, myocardial infarction, cardiac tamponade, clinically driven redilation of a failed Reducer, life-threatening arrhythmias (ventricular tachycardia (VT) or ventricular fibrillation (VF)), and respiratory failure through 30 days post-procedure, as adjudicated by the CEC.

In patients assigned to the Reducer, a CT angiogram will be performed at six months to document the patency of the device in the CS. To limit radiation exposure, this test is only performed in patients assigned to the Reducer and performed after the final CCS angina class assessment has been fulfilled to maintain binding.

### Cardiac magnetic resonance (CMR) mechanistic substudy

Patients enrolled in selected centers are offered participation in a CMR substudy primarily seeking to quantify the variation in myocardial tissue oxygenation in response to the CS Reducer implantation. Participants undergo a perfusion CMR at baseline and at six months following randomization. Primary CMR measurements include the stress-induced myocardial ischemic reactions, determined by first-pass perfusion imaging after contrast injection, and the blood oxygen level-dependent (BOLD) imaging. BOLD CMR is a noncontrast technique that allows assessment of myocardial oxygenation by utilizing the fact that deoxygenated hemoglobin acts as a paramagnetic endogenous contrast agent [21].

### Samples size and power calculation

The study has been powered to detect a difference in the proportion of patients improving ≥2 CCS angina classes at six months after the implantation. The following assumptions and hypotheses correspond to the primary objective:

A = 15% of participants in the sham control group will improve ≥2 CCS angina classes [22]

B = 40% of participants in the Reducer group will improve ≥2 CCS angina classes [13,14]

Hypotheses:

H_0_: A = B

H_1_: A ≠ B

Type I error rate = 5% (two-sided) with a Power = 80%

Drop out/lost to follow-up rate = 10%

Based on these assumptions, the sample size is 62 per group, for a total trial size of 124. These hypotheses will be tested with the Pearson chi-square test with continuity correction. The statistical analysis of the primary endpoint will be performed and presented following the intent-to-treat (ITT) principle. A second set of analysis will be performed using a per-protocol population, which will include all enrolled patients who completed the procedure and had a six-month CCS angina grade assessed. The safety population will include all randomized patients according to the actual treatment received. Patients who die from a device failure or from a cardiac death (as adjudicated by the CEC) before the six-month follow-up visit will be counted as CCS angina improvement less than two grades. A sensitivity analysis will be conducted to assess the impact of patients lost on follow-up for reasons other than cardiovascular death.

### Statistical analysis

Whenever appropriate, the mean, standard deviation (SD), median, interquartile range (IQR) will be presented for continuous variables whereas the frequencies and percentage will be calculated for categorical variables. Descriptive statistics *X*2 and Student *t* testing will be used to compare baseline and demographic characteristics between patients.

For both the dobutamine echocardiography WMSI and the exercise stress test (time to 1 mm ST-segment depression), the analysis of covariance (ANCOVA) will be used to compare the variation of the change from baseline to six months between the Reducer and the sham-implanted patients. In both cases, the baseline measurements will be adjusted for in the analysis [23]. Exercise stress test data are known to be non-normally distributed. If needed this data will be transformed or analyzed with nonparametric ANCOVA [24]. If appropriate, multivariable linear models will be used to assess the association between independent predictors and the continuous endpoints of the trial. The survival free of major cardiac events (cardiac death, major stroke, and myocardial infarction) will be displayed using Kaplan-Meier curves. Depending on the number of events recorded, multivariable Cox proportional hazards analysis will be attempted.

Prespecified subgroups will examine the effect of Reducer stratified according to: 1) the location of the myocardial ischemia, including the right coronary artery territory; 2) baseline left ventricular ejection fraction (LVEF), and; 3) the total myocardial ischemia burden. *P* values <0.05 will be considered statistically significant. Given that COSIRA is a phase II study, no type I error adjustment for multiple comparisons will be incorporated. All analyses will be performed using SAS version 9 or greater (SAS Institute, Inc, Cary, NC, USA), unless otherwise noted.

### Safety and efficacy monitoring

An independent DSMB is chartered to monitor and evaluate patient safety to identify any clinically relevant trends, and to recommend whether the study should continue. The first DSMB review to assess safety will occur after approximately 30 randomized patients have 30-day data available. After approximately 50% of the cohort has completed their six-month visit, the DSMB will review safety and the results of an interim assessment of the primary outcome. The results of the interim efficacy assessment will be based on the Lan-DeMets method using an O’Brien-Fleming sending function, with one interim evaluation. At the time of the interim assessment of the primary outcome, the DSMB will also review the results from a conditional power analysis for futility.

## Discussion

The COSIRA trial will investigate whether the percutaneous reduction of the CS will reduce angina in patients with refractory angina. By creating a controlled local narrowing in the CS, the Reducer increases the upstream coronary sinus pressure, which is thought to balance the regional capillary pressure, decrease resistance in the subendocardial capillaries, and favor a redistribution of oxygenated coronary blood toward the underperfused subendocardium. The coronary sinus Reducer potentially represents a new percutaneous treatment option for a growing population of patients with limited revascularization options and persistent angina symptoms.

The Reducer is a permanently implanted device with theoretical risks and inconvenience. The Reducer could lead to a CS thrombosis. However, data from the cardiac resynchronization therapy seem to suggest that foreign objects (such as leads) inserted in the CS rarely lead to thrombosis. When such thromboses do occur, their course is unpredictable and often clinically silent. The duplicity of collateral circulation between the great cardiac veins and the Thebesian veins can prevent myocardial injury [25]. In patients with an insufficient collateral network or with a variant anatomy, an acute coronary sinus thrombosis could be potentially fatal. Cases of venous hemorrhagic myocardial infarction have been reported, along with pericardial tamponade and sudden cardiac death [26]. In the COSIRA trial, acute Reducer thromboses are prevented by minimizing endothelial injury with the customized delivery catheter. While not proven efficient in venous circulation, dual antiplatelet therapy for a minimum of six months is also administered. In the eventuality of a Reducer thrombosis, thrombectomy can be performed [27] and if needed the narrowed hourglass neck of the Reducer can be completely expanded by angioplasty to allow an unrestricted passage of venous blood. The long-term consequences of myocardial venous hypertension remain largely unknown. We anticipate that six months should be enough to detect the adverse consequences of myocardial venous hypertension that could result from the Reducer implantation. Potential adverse consequences such as diastolic dysfunction or cardiac fibrosis should be picked up by the echocardiographic and cardiac resonance imaging follow-up. Of note, no such adverse consequences could be observed in the phase I patients followed up for more than three years [14].

The COSIRA trial is a phase II trial and several limitations inherent in this type of study must be discussed. The COSIRA trial was primarily designed to test whether the Reducer can improve symptoms of refractory angina. The relatively small sample size of COSIRA will not answer the clinical benefit associated with the Reducer based upon hard cardiovascular events. Still, development programs for devices to treat chronic symptomatic conditions do not have to unequivocally demonstrate a net clinical benefit on hard endpoints such as mortality or myocardial infarction [28]. However, we are confident that the COSIRA trial will provide the required amount of information to gauge the level of risk with respect to the anticipated symptomatic relief gained with the device. Ultimately, the information will be used to help design future clinical investigations.

We feel that patient-reported angina (CCS) complemented by the patient-centered SAQ, is the most appropriate endpoint in a phase II trial. However, angina is subject to a placebo effect, and biased by several factors despite blinding. Traditionally, time to 1 mm ST-segment deviation and other stress test outcomes are regarded by regulatory agencies as the most appropriate endpoints to approve new therapies. Consequently, many angina trials have used an exercise treadmill test or bicycle ergometer outcomes as primary endpoints. A large proportion of patients with advanced CAD (such as those targeted in the COSIRA trial) are unable to exercise appropriately. Consequently, efficacy conclusions drawn from a trial using stress test outcomes may have been poorly generalizable to a large contingent of patients who may potentially benefit from this therapy. Likewise, it would have been challenging to reach a statistically sound conclusion with the intended sample size for COSIRA given the important coefficient of variations typically observed with stress test measurements. For this reason, time to 1 mm ST-segment deviation will be monitored in the COSIRA trial but will not be used as a formal inclusion criterion. Finally, the Reducer may not address myocardial ischemia resulting from right coronary artery disease, since the territory is usually not drained by the CS. Information collected during the COSIRA trial should help answer this question.

## Trial status

At the time of manuscript submission, 70 patients have been enrolled in the trial, of which 45 have completed the six-month follow-up.

## Competing interests

Dr Henry, Dr White, and Dr Edelman have served on the scientific advisory board of Neovasc for the COSIRA trial. Dr Banai and Mr Schwartz are respectively the Medical Director and the Director of Clinical and Regulatory Affairs at Neovasc. All other authors have reported that they have no relationships relevant to the contents of this paper to disclose.

## Authors’ contributions

EMJ has participated in the conception of the study, participated in its design (including the statistical analysis plan) and coordination, recruited participants, and drafted the manuscript. SB has participated in the conception of the study and participated in its design (including the statistical analysis plan) and coordination. TDH has participated in the conception of the study. MS has participated in the conception of the study, and participated in its design and coordination. SG has participated in the design and coordination of the study, and has recruited participants. CJW has participated in the conception of the study. EE has participated in the design and coordination of the study. SV has participated in the conception of the study, participated in its design and coordination, recruited participants, and drafted the manuscript. All authors read and approved the final manuscript.

## References

[B1] Gibbons RJ Abrams J Chatterjee K Daley J Deedwania PC Douglas JS Ferguson TB Jr Fihn SD Fraker TD Jr Gardin JM O'Rourke RA Pasternak RC Williams SV ACC/AHA 2002 guideline update for the management of patients with chronic stable angina-summary article: a report of the American College of Cardiology/American Heart Association Task Force on practice guidelines (Committee on the Management of Patients With Chronic Stable Angina) J Am Coll Cardiol 2003 41 159 168 12570960 10.1016/s0735-1097(02)02848-6

[B2] Jolicoeur EM Granger CB Henry TD Stockbridge N Smith S Mark D Califf RM Henry TD Chaitman BR Granger CB Working Group Members Clinical and research issues regarding chronic advanced coronary artery disease: part I: contemporary and emerging therapies Am Heart J 2008 155 418 434 10.1016/j.ahj.2007.12.004 18294474

[B3] Williams B Menon M Satran D Hayward D Hodges JS Burke MN Johnson RK Poulose AK Traverse JH Henry TD Patients with coronary artery disease not amenable to traditional revascularization: prevalence and 3-year mortality Catheter Cardiovasc Interv 2010 75 886 891 20432394 10.1002/ccd.22431

[B4] Mukherjee D Bhatt DL Roe MT Patel V Ellis SG Direct myocardial revascularization and angiogenesis-how many patients might be eligible? Am J Cardiol 1999 84 A8 A600 10.1016/s0002-9149(99)00387-2 10482164

[B5] Paz Y Shinfeld A Mild increase in coronary sinus pressure with coronary sinus reducer stent for treatment of refractory angina Nat Clin Pract Cardiovasc Med 2009 6 E3 10.1038/ncpcardio1475 19234494

[B6] Beck CS Leighninger DS Operations for coronary artery disease J Am Med Assoc 1954 156 1226 1233 10.1001/jama.1954.02950130006002 13211223

[B7] Beck CS Leighninger DS Scientific basis for the surgical treatment of coronary artery disease J Am Med Assoc 1955 159 1264 1271 10.1001/jama.1955.02960300008003 13271060

[B8] Sandler G Slesser BV Lawson CW The Beck operation in the treatment of angina pectoris Thorax 1967 22 34 37 10.1136/thx.22.1.34 4961992 PMC471586

[B9] Wising PJ The Beck-I operation for angina pectoris: medical aspects Acta Med Scand 1963 174 93 98 14042469 10.1111/j.0954-6820.1963.tb07895.x

[B10] Brofman BL Medical evaluation of the Beck operation for coronary artery disease J Am Med Assoc 1956 162 1603 1606 10.1001/jama.1956.02970350019005 13376326

[B11] Camici PG Crea F Coronary microvascular dysfunction N Engl J Med 2007 356 830 840 10.1056/NEJMra061889 17314342

[B12] Ido A Hasebe N Matsuhashi H Kikuchi K Coronary sinus occlusion enhances coronary collateral flow and reduces subendocardial ischemia Am J Physiol Heart Circ Physiol 2001 280 H1361 H1367 11179085 10.1152/ajpheart.2001.280.3.H1361

[B13] Banai S Ben MS Parikh KH Medina A Sievert H Seth A Tsehori J Paz Y Sheinfeld A Keren G Coronary sinus reducer stent for the treatment of chronic refractory angina pectoris: a prospective, open-label, multicenter, safety feasibility first-in-man study J Am Coll Cardiol 2007 49 1783 1789 10.1016/j.jacc.2007.01.061 17466229

[B14] Banai S Schwart M Sievert H Seth A Keren G Parikh KH Long-term follow-up to evaluate the safety of the neovasc reducer, a device-based therapy for chronic refractory angina J Am Coll Cardiol 2010 55 A98.E927 10.1016/S0735-1097(10)60928-X

[B15] McGillion M Arthur HM Cook A Carroll SL Victor JC L'allier PL Jolicoeur EM Svorkdal N Niznick J Teoh K Cosman T Sessle B Watt-Watson J Clark A Taenzer P Coyte P Malysh L Galte C Stone J Management of patients with refractory angina: Canadian cardiovascular society/Canadian pain society joint guidelines Can J Cardiol 2012 Suppl 2 S20 S41 22424281 10.1016/j.cjca.2011.07.007

[B16] Jolicoeur EM Cartier R Henry TD Barsness GW Bourassa MG McGillion M L'Allier PL Patients with coronary artery disease unsuitable for revascularization: definition, general principles, and a classification Can J Cardiol 2012 Suppl 2 S50 S59 22424284 10.1016/j.cjca.2011.10.015

[B17] Moher D Schulz KF Altman DG The CONSORT statement: revised recommendations for improving the quality of reports of parallel-group randomized trials Ann Intern Med 2001 134 657 662 11304106 10.7326/0003-4819-134-8-200104170-00011

[B18] Chaitman BR Stone PH Knatterud GL Forman SA Sopko G Bourassa MG Pratt C Rogers WJ Pepine CJ Conti CR The ACIP Investigators Asymptomatic Cardiac Ischemia Pilot (ACIP) study: impact of anti-ischemia therapy on 12-week rest electrocardiogram and exercise test outcomes J Am Coll Cardiol 1995 26 585 593 10.1016/0735-1097(95)00013-T 7642847

[B19] Frolicher VF Myers J Manual of Exercise Testing 3rd edition 2007 Philadelphia: Mosby Elsevier

[B20] Spertus JA Winder JA Dewhurst TA Deyo RA Prodzinski J McDonell M Fihn Development and evaluation of the Seattle Angina questionnaire: a new functional status measure for coronary artery disease J Am Coll Cardiol 1995 25 333 341 10.1016/0735-1097(94)00397-9 7829785

[B21] Friedrich MG Niendorf T Schulz-Menger J Gross CM Dietz R Blood oxygen level-dependent magnetic resonance imaging in patients with stress-induced angina Circulation 2003 108 2219 2223 10.1161/01.CIR.0000095271.08248.EA 14557359

[B22] Losordo DW Henry TD Davidson C Sup Lee J Costa MA Bass T Mendelsohn F Fortuin FD Pepine CJ Traverse JH Amrani D Ewenstein BM Riedel N Story K Barker K Povsic TJ Harrington RA Schatz RA ACT34-CMI Investigators Intramyocardial, autologous CD34+ cell therapy for refractory angina Circ Res 2011 109 428 436 10.1161/CIRCRESAHA.111.245993 21737787 PMC3190575

[B23] Vickers AJ Altman DG Statistics notes: analysing controlled trials with baseline and follow up measurements BMJ 2001 323 1123 1124 10.1136/bmj.323.7321.1123 11701584 PMC1121605

[B24] Knoke JD Nonparametric analysis of covariance for comparing change in randomized studies with baseline values subject to error Biometrics 1991 47 523 533 10.2307/2532143 1912259

[B25] Von LM The venous drainage of the human myocardium Adv Anat Embryol Cell Biol 2003 168 1 104 10.1007/978-3-642-55623-4_1 12645157

[B26] Parmar RC Kulkarni S Nayar S Shivaraman A Coronary sinus thrombosis J Postgrad Med 2002 48 312 313 12571393

[B27] Neri E Tripodi A Tucci E Capannini G Sassi C Dramatic improvement of LV function after coronary sinus thromboembolectomy Ann Thorac Surg 2000 70 961 963 10.1016/S0003-4975(00)01639-8 11016343

[B28] Jolicoeur EM Ohman EM Temple R Swain J Stockbridge N Witten C Smith S Mark D Califf RM Henry TD Chaitman BR Granger CB Clinical and research issues regarding chronic advanced coronary artery disease part II: trial design, outcomes, and regulatory issues Am Heart J 2008 155 435 444 10.1016/j.ahj.2007.12.005 18294475

